# Does human support add value to persuasive design-based digital mental health interventions? A propensity score matching study of a digital parenting program

**DOI:** 10.3389/fdgth.2025.1586668

**Published:** 2025-12-02

**Authors:** Chen R. Saar, Or Brandes, Amit Baumel

**Affiliations:** Department of Community Mental Health, University of Haifa, Haifa, Israel

**Keywords:** human support, persuasive design, user engagement, parent training, mental health, child behavior problems

## Abstract

**Background:**

While human support has been shown to increase user engagement with digital mental health interventions, it also increases managerial overhead, raises costs, and limits scalability. An alternative approach leverages persuasive design principles to potentially reduce the reliance on human support. Therapeutic persuasiveness (TP) is a concept for persuasive design that involves incorporating features that encourage users to make positive behavior changes in their lives. Prior research suggests that TP features can effectively improve both user engagement and intervention outcomes.

**Objective:**

This study aimed to evaluate the added value of human support in a TP-enhanced digital parent training program (DPT) by comparing engagement and clinical outcomes between human-supported and self-directed intervention formats.

**Methods:**

A propensity score matching approach was used to utilize data from two comparable studies, involving parents of children aged 3–7, all of whom received the same TP-enhanced DPT. One study included a self-directed condition (*n* = 38), while the other included a human-supported condition (*n* = 38). Human support was provided via chat and phone calls and included progress acknowledgments, personalized feedback, disengagement follow-up, and timely responses to parent-initiated messages. Engagement patterns and pre-to-post intervention changes in child behavior, parenting practices, and parental self-efficacy were compared between the two intervention formats.

**Results:**

There were no significant differences between the self-directed and human-supported formats in program completion rates (89% vs. 92%, respectively; *P* = .51), the percentage of parents completing all the modules (81.6% vs. 76.3, *P* = .57) or total usage time (137 vs. 141 min, *P* = .14). Parents in the human-supported version logged in significantly more frequently than those in the self-directed group (Cohen's *d_s_* = 0.32, 0.34; *P_s_* ≤ .04), which is attributed to parents’ additional engagement in messaging with the supporter. No significant differences were observed between groups in reported improvements in children's behavior problems, parenting practices, or parental self-efficacy (*P_s_* ≥ .17).

**Conclusions:**

These findings suggest that well-designed, technology-enabled intervention features may effectively support program adherence and therapeutic outcomes without requiring additional human support. This study highlights the importance of further research into the relative impact of human-supported vs. self-directed DMHIs and investigating how intervention quality might influence this impact.

## Introduction

Digital mental health interventions (DMHI) have the potential to expand access to evidence-based mental health care at affordable rates (1–3). However, a major challenge in developing effective DMHIs lies in maintaining user engagement over time and ensuring adherence to the therapeutic process as intended (4–6).

One well-studied approach to improving engagement with DMHIs is the inclusion of human support, delivered by a trained clinician or a coach via chat, phone or email. The mode of support can vary, ranging from simple reminders to complete activities to full therapeutic sessions conducted alongside the DMHI (7). According to the *Efficiency Model of Support* (8), support encompasses three distinct areas designed to address specific failure points: technical support (addressing usability failures), use support (addressing engagement failures), and clinical support (ensuring that skills and knowledge are effectively applied in daily life). The supporter may receive information from the user or the system and tailor the guidance to meet the user's specific needs at a given time. While some reviews have concluded that human support improves engagement and outcomes [e.g., (3, 9, 10)], others have been less conclusive [e.g., (11)]. In either case, incorporating human support increases the costs of DMHIs delivery, ultimately limiting scalability and accessibility.

A promising approach to maintaining user engagement in self-directed DMHIs is enhancing intervention quality through persuasive system design, which aims to seamlessly tunnel users through the therapeutic process (12). This design approach has been shown to enhance user engagement (13–15). One example of such design approach is enhancing the program's “therapeutic persuasiveness” (TP), which refers to the extent to which an intervention's features are designed to encourage users to make positive behavior changes in their lives (16). TP features, such as call to action prompts, task monitoring with feedback, and adaptation to user's state, have been found to predict program usage in real-world use of web-based behavior interventions, accounting for 11%–42% of the variance in program usage within the regression models (5, 17).

Building on these findings, empirical research has investigated whether directly manipulating TP levels impacts DHMI usage and efficacy. A pilot randomized controlled trial (RCT) (16) evaluated the impact of TP features on engagement and outcomes in a digital parent training program (DPT). Parents using a TP-enhanced version demonstrated significantly higher engagement (68.9% vs. 27.9% of parents completed all program modules) and greater improvements in child behavior (Cohen's *d_s_* = 0.43, 0.54) compared to those using a basic DPT version. These results raise the following question: Would adding human support to the TP-enhanced version further improve engagement and outcomes, or are TP features alone sufficient to facilitate the therapeutic process, potentially reducing the need for human support?

To address this question, the present study employed a propensity score matching paradigm to compare engagement and outcomes between parents using the TP-enhanced version with and without human support. The analysis utilized two datasets: data from parents who used the self-directed (unguided) version (U-DPT), collected in a previous pilot study (16), and data from parents using the human-supported version (S-DPT), collected in a subsequent follow-up study. Both studies implemented the same DPT, similar recruitment processes and parent populations, with the incorporation of human support being the primary distinguishing factor. The primary objectives of the current study were to estimate effect size differences and evaluate the added value of human support in enhancing engagement and outcomes.

## Methods

### Study design

This study utilized two datasets collected from separate trials examining a TP-enhanced DPT, implemented with and without human support. The first dataset originated from an RCT comparing a TP-enhanced to a basic version of the DPT (16). The second dataset was obtained from a subsequent study examining a comparable TP-enhanced version that included human support. Both studies employed automated system tracking for usage data collection and gathered outcome measures through self-reported questionnaires administered via Qualtrics at baseline and post-intervention (10 weeks from baseline).

### Participants and recruitment procedure

Study protocols were approved by the institutional review board of the University of Haifa (approval numbers: 058/22 for the self-directed intervention; 418/23 for the human-supported intervention). No financial or material incentives were provided for parents to encourage their use of the digital program.

The eligibility criteria for participating parents were: having a child aged 3–7 with an elevated level of behavior problems, and access to a smartphone with an internet connection. Exclusion criteria were: child receiving treatment for behavioral/emotional difficulties or parent enrolled in another parent training program, and child diagnosed with an intellectual disability or developmental delay.

Participants for the first study were recruited between May and July 2022, and for the second study between January 2023 and January 2024, using similar Facebook advertising campaign, targeting parents of children aged 4–8 residing in Israel, without focusing on specific platform subgroups (e.g., parenting groups). The campaign targeted Hebrew-speaking parents, as the program and all study materials were provided in Hebrew. The advertisement consisted of a picture of a parent and children accompanied by a short paragraph outlining the study's aim and the eligibility criteria. Parents who expressed interest were directed to follow a link to the study's web page, where they provided their contact details and completed a preliminary eligibility questionnaire based on exclusion criteria and items related to child behavior. Parents who met the preliminary eligibility criteria were contacted by phone to confirm their eligibility, interest, and understanding of study terms. Interested parents signed a web-based consent form and completed a baseline assessment. Eligibility was then confirmed using Eyberg Child Behavior Inventory (ECBI) subscale scores (ECBI-Problem ≥ 15 or ECBI-Intensity ≥ 132). In the second study, eligibility was further assessed through a remote video interview with an independent clinician (OB), based on the Oppositional Defiant Disorder (ODD) criteria from the Diagnostic and Statistical Manual of Mental Disorders, Fifth Edition (DSM-5). In total, 45 parents met the eligibility criteria in the first study and were provided access to the U-DPT, while 55 parents met the criteria in the second study and received access to the S-DPT. Six parents dropped out of the first study (6/45, 13.3%; four lost to follow-up, one discontinued participation, and one withdrew due to a medical reason), and seven parents dropped out of the second study (7/55, 12.73%; five lost to follow-up and two discontinued participation). A total of 39 parents who used the U-DPT and 48 parents who used the S-DPT completed the post-intervention assessment and were included in propensity score matching to balance ECBI levels at pre-intervention.

### Overview of benevolent parenting intervention

**Figure 1:**
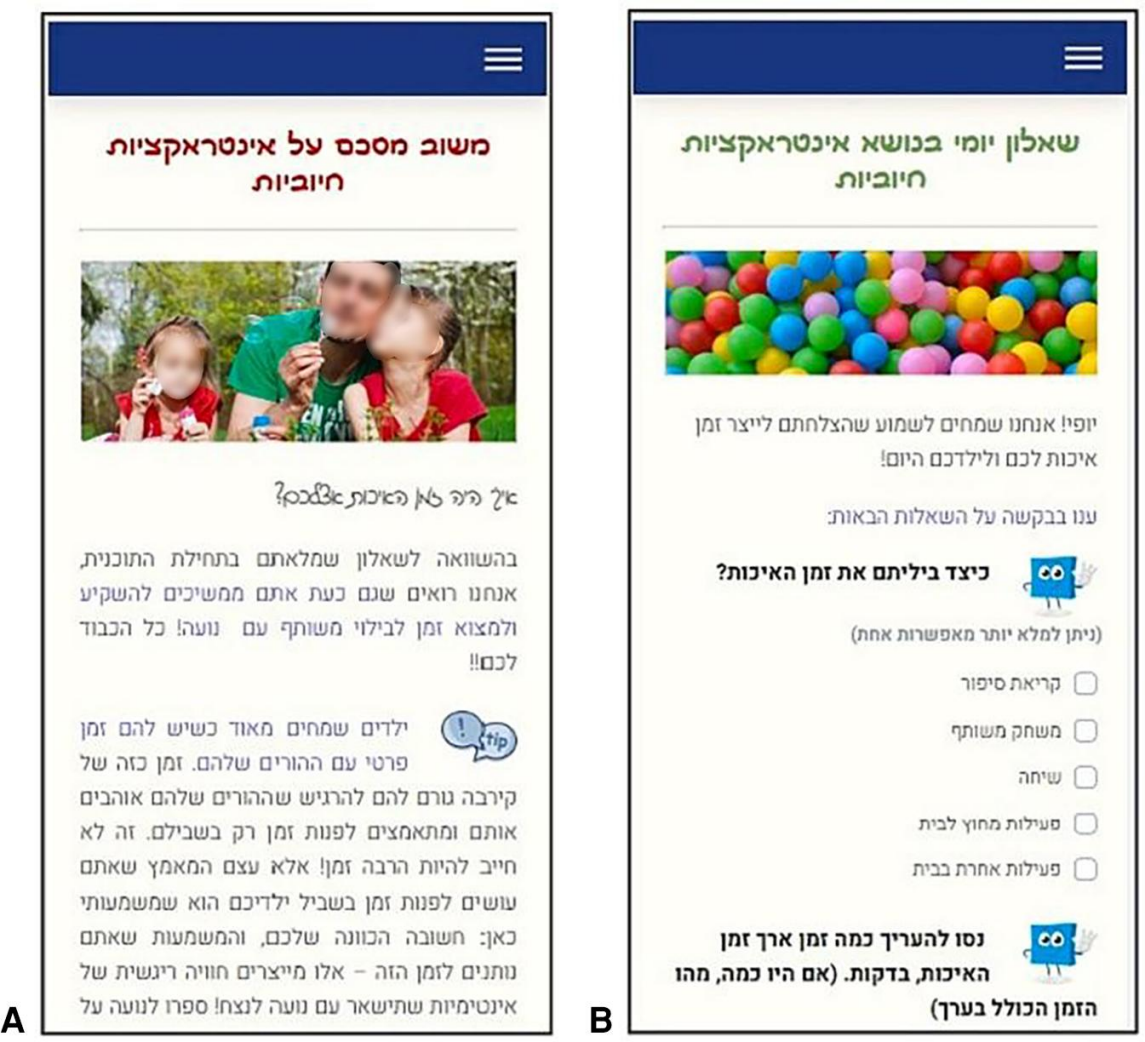
Mobile screenshots of the daily monitoring questionnaire **(A)** and the personalized positive feedback message [**(B)**; text in Hebrew]. Images/icons reproduced from Pixabay (https://pixabay.com/): “Baby Dad Daughter” by ua_Bob_Dmyt_ua, “Dialog Tip” by OpenClipart-Vectors, “Balls Children's Playground” by palichka and “Square Shape” by GraphicMama-team, licensed under Content License.

The “Benevolent Parenting” program is based on the principle that parental behaviors and reactions significantly influence a child's behavior (18, 19). The program protocol, designed for completion within two months, was developed by Prof. Baumel, and integrates evidence-based components utilized in common parent training programs and includes seven modules: (1) introduction to parent training; (2) positive interactions and quality time; (3) parental emotion regulation; (4) effective routines and clear ground rules; (5) recognizing positive behaviors and ignoring minor negative behaviors; (6) overcoming disobedience; and (7) mindful parenting and communication between partners (16, 20). Each module began with a *learning phase* (10–25 min), presenting core concepts through videos, texts, and interactive features, including multiple-choice questions providing immediate feedback and responses to frequently asked questions. Each learning phase concluded with recommendations for practicing the acquired skills. To support skill acquisition, each learning phase was followed by a *focusing phase* (1–2 week) designed to increase the salience of therapeutic activities in parents' daily lives and facilitate skill development in a nonjudgmental manner. Accordingly, the following TP features were integrated into the focusing phase:

1. Call-to-action messages: Text messages with tips and motivating messages aligned with the relevant module were sent just before childcare pickup hours. For example, parents focusing on “positive interactions and quality time” received motivational messages with ideas for fun activities. Additionally, reminders were sent to re-engage parents who missed daily questionnaires or failed to complete a learning phase within a week. These digital triggers were designed and tailored based on the principles by Muench & Baumel (21).
2. Monitoring and ongoing feedback: Short daily questionnaires assessed parents’ application of newly acquired skills, with automatic, nonjudgmental feedback provided daily and weekly (see Figure 1). The questions were based on the relevant theme being practiced and were designed to help parents effortlessly integrate the associated techniques (22). For example, parents focusing on “effective routines” were asked if they had followed the relevant guidelines with their children that day.
3. Adaptation to user state: Parents could personalize the program based on their responses to questionnaires, with optional modules recommended to address specific needs.

Program content was created in a content management system that enabled delivery of web-based content, text messages, and email, all triggered by logic-based rules (if–then statements). The program was accessible through a mobile- and computer-compatible website and was delivered via MindTools, an open-source eHealth platform initially developed under the name Serafin. The platform was subsequently adapted and upgraded by the last author (AB) and is available on GitHub (23).

### Human support

For the guided DPT (G-DPT), human support followed a coaching manual based on previous work (24, 25) as well as the *supportive accountability model* and the *efficiency model of support* (8, 26). The support framework aimed to enhance adherence to the therapeutic process through encouraging program utilization, facilitating skill practice, and providing guidance and problem-solving support.

The supporter was a licensed educational psychologist trained and supervised by the study's PI. Support was provided via a chat feature embedded in the DPT and through phone calls. Support included: (1) initiating contact after the first login to set expectations and offer an optional phone call; (2) sending messages after each learning phase to summarize progress and introduce the upcoming focusing phase; (3) acknowledging efforts during the focusing phase and providing tips and information based on daily questionnaires; (4) offering a phone call after the “overcoming disobedience” phase to clarify key points. To address disengagement (e.g., failing to complete a learning phase within a week or respond to the daily questionnaire for five consecutive days), the supporter contacted the parent through the chat to offer additional support. Furthermore, the supporter responded to all parent-initiated messages within one working day.

Data on user engagement with the human-supporter in the guided version of the program is reported extensively in another paper that is currently under review. Here, we provide only succinct details for brevity, as they may be helpful for the comparisons made in this paper: Parents sent an average of 6.18 messages (SD = 5.93) to the supporter and spent an average of 8.60 min (SD = 11.83) on phone calls with them.

### Measures

Study data consisted of self-reported pre- and post-intervention (10 weeks) questionnaires from parents, as well as program usage metrics. Parents completed questionnaires using Qualtrics.

#### Eyberg child behavior inventory (ECBI)

Children's behavior problems were assessed using two subscales of the ECBI: Intensity and Problem (27). For each of the 36 behaviors, parents rated the intensity on a Likert scale from 1 (never) to 7 (always) and indicated whether the behavior was a problem (0 = no; 1 = yes) (28, 29). The internal consistency in this study (Cronbach's alpha) was .90 for ECBI-Problem and .91 for ECBI-Intensity.

#### The parenting scale (PS)

The PS (30) assesses parental responses to their child's misbehavior, offering both effective and ineffective options for each hypothetical situation. Parents rated their typical response on a 7-point Likert scale (7 = ineffective; 1 = effective). Two subscales of the PS were used: overreactivity (11 items) and laxness (10 items). Internal consistency was acceptable, with Cronbach's alpha coefficients of .80 for overreactivity, and .89 for laxness.

#### The parenting tasks checklist (PTC)

The PTC (31) evaluates task-specific self-efficacy, measuring parents' confidence in managing parenting tasks and situations. A shorter version was used for this study, comprising two subscales aligned with the full version: behavioral self-efficacy (6 items, e.g., “Throws a tantrum”) and setting self-efficacy (6 items, e.g., “Shopping with child”). Parents rated each item on a scale from 0 (“Certain I can't do it”) to 100 (“Certain I can do it”). Internal consistency was high with Cronbach's alphas scores of .89 for the setting self-efficacy, and .96 for the behavior self-efficacy.

#### Alabama parenting questionnaire (APQ) positive parenting practices

Positive parenting practices were evaluated using the APQ Positive Parenting Practices subscale (32), which includes 3 items (e.g., “You make sure your child knows when he or she has done something good”). Parents rated each item on a 5-point Likert scale from 1 (never) to 5 (always), reflecting the frequency of these behaviors at home. The internal consistency score in this study was .88.

#### Ehealth therapeutic alliance inventory (ETAI)

The ETAI (33) assesses the therapeutic alliance in eHealth interventions using 14 items describing parents' thoughts or feelings towards the DPT (e.g., “Benevolent Parenting focuses on goals that are important to me and helps me achieve them”). Parents rated their level of agreement with each statement on a 7-point Likert scale (1 = Strongly Disagree; 7 = Strongly Agree). The internal consistency score of the scale was .87.

#### Acceptance (satisfaction)

Parents' attitudes and satisfaction with the DPT were assessed post-intervention. Perceived usefulness (e.g., “The DPT helped me manage my child's misbehavior/become a better parent”) and satisfaction (“I would recommend the DPT to parents of children with behavior problems”) were evaluated using a 5-point Likert scale from 1 (I do not agree) to 5 (I agree). A similar scale has been successfully used in previous studies (1, 24).

#### Program usage and completion rate

Program usage metrics were collected automatically and included the number of login days, unique logins, total usage time (in minutes), and total time spent using the program (in weeks, from first to last login). Program completion metrics included the percentage of parents who completed the “overcoming disobedience” module (the main obligatory component of the program), percentage of parents who completed the entire program, and completion ratio (number of modules completed divided by the total number of modules in the program).

### Statistical analyzes

Descriptive statistics were used to analyze demographic parameters, program usage metrics, and child and parent outcomes. Differences in continuous variables were analyzed using paired-sample t-tests. Differences in dichotomous variables were analyzed using McNemar's test (34).

#### Comparison of U-DPT vs. S-DPT using propensity score matching

As the U-DPT and the S-DPT studies were conducted in different time periods, it was necessary to control for potential confounders between the two groups. A propensity score matching approach was applied to balance the following covariates: child's age and gender, number of participating parents (e.g., single or both parents), family income, and ECBI scores at pre-assessment (35, 36). Propensity scores were calculated for U-DPT (*n* = 39) and the S-DPT (*n* = 48) participants. Pairs were matched using a recommended match tolerance (caliper) of 0.12 (37), selecting the most similar propensity score without replacement. Baseline variables balance was assessed using standardized bias, defined as the mean difference divided by the common standard deviation. Standardized biases for continuous variables were considered acceptable at ≤0.2 and for binary variables at ≤0.1 (37, 38). Statistical significance was defined as *p* < .05. Exact *p*-values are reported for all analyses to ensure transparency. All statistical analyses were conducted using IBM SPSS Statistics, version 27.

## Results

Overall, 38 pairs of parents met the recommended match tolerance for the propensity scores matching paradigm and were included in the subsequent analysis. Descriptive statistics for parents' socio-demographic characteristics by DPT group are presented in Table 1. The average age of the children was 4.95 years; the average age of the parents was 36.19 years. On average, families had 2.62 children. To reflect the Israeli population and cultural context, household income was reported in Israeli Shekels [ILS; the average household monthly income was approx. 16,500 ILS in 2023, (39)], and religiosity, a salient cultural factor of Israeli population, was included among the demographic characteristics. No significant differences were found in demographic characteristics between the two study groups.

**Table 1:** Participant demographic characteristics by intervention group at baseline.

Categorical	Variable	U-DPT *N* = 38	S-DPT *N* = 38	*χ^2^* (1)	*P*
		*N* (%)	*N* (%)		
Child gender	Male	22 (57.9%)	21 (55.3%)	0.05	.82
	Female	16 (42.1%)	17 (44.7%)		
Number of parents	Both parents	23 (60.5%)	21 (55.3%)	0.22	.64
	One parent	15 (39.5%)	17 (44.7%)		
Education^a^	Secondary education	4 (10.5%)	7 (18.4%)	0.96	.33
	Post-secondary education	34 (89.5%)	31 (81.6%)		
Religiosity	Secular	22 (57.9%)	20 (52.6%)	1.15	.77
	Traditional	9 (23.7%)	10 (26.3%)		
	Religious	7 (18.4%)	8 (21%)		
Hours of work/study per week^a^	Under 30	12 (31.6%)	10 (26.3%)	1.75	.42
	Between 30 and 39	7 (18.4%)	12 (31.6%)		
	More than 39	19 (50%)	16 (42.1%)		
House level income^b^	<15,000	6 (15.8%)	10 (26.3%)	3.56	.17
	15,000–18,000	14 (36.8%)	7 (18.4%)		
	> 18,000	18 (47.4%)	21 (55.3%)		
Continuous		*M* (SD)	*M* (SD)	*T (37)*	*P*
Parent age (years)^a^		36.42 (3.31)	35.81 (3.42)	0.88	.38
Child age (years)		4.87 (1.24)	5.03 (1.09)	−0.65	.52
Number of children in family		2.76 (1.00)	2.47 (0.92)	1.19	.24

a Refers to the parent leading the intervention;

b In Israeli Shekel (ILS).

Program usage and completion rates by study groups are presented in Table 2. The program completion ratio was .89 for the U-DPT group and .92 for the S-DPT group. No significant differences were observed in the percentage of parents completing the “overcoming disobedience” module (the critical module for addressing behavior problems) or in the percentage of parents completing all modules in their personalized program. However, significant differences were found in the number of login days and unique logins, with S-DPT users logging in more frequently.

**Table 2:** Program completion and usage metrics.

Program usage	U-DPT *N* = 38	S-DPT *N* = 38			
Dichotomous	% (*n*/*n*)	% (*n*/*n*)	*χ^2^* (1)	*P*	
“overcoming disobedience” module completers	33 (86.8%)	34 (89.5%)	0.13	.72	
Program completers	31 (81.6%)	29 (76.3%)	0.32	.57	
Continuous	*M* (SD)	*M* (SD)	*t* (df = 37)	*P*	Cohen's *d*
Completion ratio^a^	0.89 (0.25)	0.92 (0.15)	−0.67	.51	−0.11
Number of login days	23.10 (9.39)	27.87 (10.92)	−1.97	.**05**	**−0**.**32**
Unique logins	25.44 (11.07)	31.58 (12.99)	−2.11	.**04**	**−0**.**34**
Usage time (minutes)	137.13 (72.83)	141.39 (44.29)	−0.28	.78	−0.04
Time using the program (weeks)	10.84 (5.10)	12.61 (5.03)	−1.51	.14	−0.24

Significant *p* values (*p* ≤ .05) and their corresponding Cohen's d effect sizes are shown in bold.

a Refers to the number of completed modules divided by the total number of modules in the participant personal program.

Differences between self-directed and the human-supported DPT in reported changes following the intervention are reported in Table 3. Both intervention conditions showed significant improvements from pre- to post-intervention in ECBI metrics (*P*_s_ ≤ .001) and in parenting measures (.01 ≤ *P*_s_ ≤ .001), except for the APQ. However, no significant differences were observed between the U-DPT and S-DPT groups in reported changes.

**Table 3:** Children and parents’ outcomes, between baseline and post-intervention time points.

Measurement	U-DPT *N* = 38		S-DPT *N* = 38		*t* (37)	*P*	Cohen's *d*
	Baseline *M* (SD)	Post-intervention *M* (SD)	Baseline *M* (SD)	Post-intervention *M* (SD)			
ECBI Intensity	157.58 (21.24)	119.89 (27.28)***	162.13 (21.35)	132.66 (27.76)***	−1.09	.28	−0.18
ECBI Problems	22.31 (5.40)	13.66 (1.33)***	24.21 (0.76)	16.00 (1.38)***	−0.24	.81	−0.04
PS Laxness	3.29 (0.94)	2.60 (0.91)***	3.41 (1.14)	2.88 (1.06)**	−0.65	.52	−0.10
PS Over-reactivity	3.51 (0.92)	2.76 (0.58)***	3.57 (0.67)	2.72 (1.04)***	0.56	.58	0.09
PTC setting	69.03 (23.24)	79.81 (15.60)**	65.28 (14.20)	74.51 (18.04)**	0.40	.69	0.07
PTC behavior	55.19 (24.48)	74.10 (17.95)***	48.26 (22.93)	64.69 (25.11)***	0.40	.69	0.06
APQ	12.81 (2.42)	13.47 (1.55)	4.37 (0.40)	4.49 (0.40)	1.39	.17	0.22

When an asterisk is presented at post-intervention, a significant difference between pre- and post-intervention was found using a paired sample t-test:

** Significant at *P* < .01;

*** Significant at *P* < .001.

Differences in parents' perceptions of the therapeutic alliance and program satisfaction are reported in Table 4. No significant differences were observed between the U-DPT and S-DPT groups.

**Table 4:** Reported therapeutic alliance and program satisfaction at post-intervention.

Measurement	U-DPT *N* = 38	S-DPT *N* = 38	*t* (df = 37)	*P*	Cohen's *d*
	*M* (SD)	*M* (SD)			
eHealth Therapeutic Alliance Inventory	5.15 (0.96)	5.14 (0.83)	0.04^a^	.97	0.01
Program Satisfaction					
“The DPT helped me manage my child's misbehavior”	3.72 (0.93)	3.65 (0.82)	0.33	.74	0.05
“The DPT helped me become a better parent”	3.84 (0.82)	3.89 (0.77)	−0.24	.81	−0.04
“I would recommend the DPT to parents of children with behavior problems”	4.16 (1.04)	4.14 (.86)	0.11	.91	0.02

a Reporting on 37 pairs (df = 36) of parents as one parent response was missing.

## Discussion

This study used propensity score matching to compare outcomes between two delivery formats of a DPT: a self-directed and a human-supported version, both incorporating TP features designed to support user adherence through the therapeutic process. The findings revealed comparable engagement patterns, including program completion rates and overall usage time, as well as similar improvements in children's behavior problems, parental behaviors, and parental self-efficacy. Additionally, therapeutic alliance and satisfaction levels were similar among both groups. These results align with prior research suggesting that DMHIs can achieve similar levels of adherence and efficacy regardless of whether they are delivered with or without human support (12, 40–42).

However, a significant difference in login frequency was observed, with S-DPT users logging in more frequently than U-DPT users (27.87 vs. 23.10 login days, respectively). This difference may be attributed to parent engagement in messaging with the supporter (as S-DPT participants sent an average of 6.18 messages), which likely influenced their overall login patterns.

A key factor underlying these findings may be the integration of TP features in both versions of the DPT. These features might have influenced parents' adherence by enhancing accountability toward the therapeutic process (26, 43), and by addressing specific failure points, such as low engagement and inadequate implementation of knowledge and tools (8). According to Mohr et al. (26), accountability is strengthened when clear, process-oriented expectations are set, prompting users to justify their actions. In this study, TP features, including daily call-to-action messages and automated feedback following daily questionnaires, could have fostered accountability by building anticipation and ensuring continuous interaction between parents and the program while addressing common implementation barriers. The presence of clear expectations, monitoring, and feedback through TP features may explain the similar adherence patterns observed across both groups, suggesting that the human support protocol (as implemented in this study) may have been redundant. This finding also supports prior propositions that adequate product design can positively impact user adherence (5, 44).

Another related dimension is the therapeutic alliance, measured using the ETAI, which assesses application-induced accountability alongside sense of relatedness to the intervention, perceived emotional investment, and the traditional aspects of the therapeutic alliance nurtured in client–therapist collaboration (11, 45). Therapeutic alliance has been suggested to predict engagement in both human-supported and self-directed DMHIs (46). The similarity in therapeutic alliance scores across both conditions suggests that the limited human support protocol employed (e.g., mostly through chat messages and an average of less than nine minutes of phone calls per parent) did not uniquely enhanced therapeutic alliance, engagement, or outcomes. This finding further highlights the capacity of well-designed automated features to replicate some aspects of human interaction, thereby reducing the necessity for resource-intensive human support.

### Limitations and future directions

This study has several limitations that should be noted. First, as a quasi-experiment study with a limited sample size, it lacks both the statistical power and the randomization of participants that a full scale RCT provides. Conducting an RCT of the TP-enhanced DPT, with and without human support, on a larger sample, would allow for a more nuanced examination of the effects of DMHI design quality on the added value of human support. Moreover, the nature of recruitment via social networks introduces the possibility of self-selection bias, which may limit the representativeness of the sample relative to the broader Israeli parent population. Nevertheless, previous research has shown that social media recruitment, particularly through Facebook, can actually reach broader and more diverse populations across socioeconomic backgrounds (47). In line with this, the current sample included participants from diverse income levels and varying degrees of religiosity.

Second, the specific human support protocol used in this study (e.g., its method, frequency, and intensity) may have influenced the results. Prior research suggests that variations in support protocols can significantly impact adherence and outcomes [e.g., (48, 49)]. To generalize the findings, future studies should investigate a broader range of support models to determine which protocols provide the greatest value for money in terms of adherence and outcomes, considering the target populations and therapeutic goals. Third, this study focused on a DMHI designed to support children through their parents. Therefore, further research is needed to determine whether similar findings apply to DMHIs that support adults with various mental health conditions. Finally, future studies could explore the integration of artificial intelligence (AI) and machine learning (ML) into self-directed DMHIs (50). These technologies could facilitate real-time, tailored, and personalized content delivery, addressing users' specific needs while eliminating the cost associated with human support.

## Conclusions

This study underscores the importance of investigating the impact of human support vs. self-directed DMHIs on engagement and outcomes, as well as the role of DMHI quality in this context. The findings contribute to the growing body of literature suggesting that well-designed, technology-enabled features may facilitate adherence and enhance therapeutic outcomes. Future research should further investigate how intervention quality influences user engagement and the extent to which human support remains necessary in different contexts.

## Data Availability

The datasets presented in this article are not readily available because these datasets are part of a larger ongoing research project. They can be made available upon specific individual requests. Requests to access the datasets should be directed to abaumel@welfare.haifa.ac.il.
